# Sinonasal mucosal melanoma treatment response assessment to immune checkpoint inhibitors using hybrid positron emission tomography imaging

**DOI:** 10.1038/s41598-023-45705-z

**Published:** 2023-11-01

**Authors:** Alexander Maurer, Nathalie A. Gstrein, Florentia Dimitriou, Thomas Sartoretti, Jan A. Schaab, Esmée L. Looman, Panagiotis Balermpas, Niels J. Rupp, Sandra N. Freiberger, Michael B. Soyka, David Holzmann, Tina Mauthe, Simon A. Mueller, Stephan Beintner-Skawran, Michael Messerli, David Kenkel, Martin W. Huellner, Christian M. Meerwein

**Affiliations:** 1 Department of Nuclear Medicine, University Hospital Zurich, University of Zurich, Zurich, Switzerland; 2 Department of Otorhinolaryngology, Head and Neck Surgery, University Hospital Zurich, University of Zurich, Frauenklinikstrasse 24, 8091 Zurich, Switzerland; 3 Department of Dermatology, University Hospital Zurich, University of Zurich, Zurich, Switzerland; 4 Department of Radiation Oncology, University Hospital Zurich, University of Zurich, Zurich, Switzerland; 5 Department of Molecular Pathology, University Hospital Zurich, University of Zurich, Zurich, Switzerland

**Keywords:** Head and neck cancer, Cancer immunotherapy, Cancer imaging, Metastasis

## Abstract

The purpose of this retrospective study was to investigate response of sinonasal mucosal melanoma (SMM) patients to treatment with immune checkpoint inhibitors (ICI), using hybrid PET imaging. Fifteen SMM patients underwent hybrid PET imaging before and three months after initiation of ICI. The disease-specific survival (DSS) was calculated. Quantitative PET parameters of the primary tumor and their association with DSS and therapy response were investigated. Nine of the fifteen (60%) patients responded to ICI therapy. Patients with therapy response depicted on hybrid PET imaging had better DSS than those without (*p* = 0.0058). Quantitative PET parameters of the initial PET harbored no association with DSS or therapy response. However, these findings lack of sufficient statistical power and must be interpreted with caution. The first restaging PET-imaging after ICI initiation can help stratify patients with regard to DSS.

## Introduction

Sinonasal mucosal melanoma (SMM) is a rare malignancy with a poor prognosis, exhibiting unpredictable biological behavior, frequent local or regional recurrence and a high metastatic potential^1–3^. Previous data indicated a 5 year-overall survival (OS) of approximately 30–40%^4^. The entity accounts for 4–7% of all sinonasal malignancies, and for 0.4–1.3% of all malignant melanomas^5,6^.

Transnasal-endoscopic tumor resection followed by postoperative radiation therapy (RT) is the gold standard of treatment^4,7,8,9^. Before the introduction of immune checkpoint inhibitors (ICI), patients with distant metastases (DM) were treated with traditional chemotherapy protocols in the first-line setting^1,10^. However, studies failed to show a clear benefit from chemotherapy with regard to OS^11^. The introduction of ICI, such as anti-CTLA-4 (ipilimumab) and anti-PD-1 antibodies (nivolumab and pembrolizumab) has revolutionized the treatment of cutaneous malignant melanoma (CMM), markedly improving OS^8,12^. Nevertheless, data on the efficacy of ICI in mucosal melanoma are scarce and no randomized clinical trials exist, owing to its rarity. Available data from a pooled analysis from clinical trials indicate that efficacy outcomes seem to be poorer in mucosal melanoma compared to CMM, with lower response rates and shorter survival^13^. These data underline the need for additional prospective studies and biomarker analysis in this rare melanoma subtype. Recently, the combined expression patterns of three tumor testis antigens have been proposed as potential predictive biomarkers in mucosal melanomas responding to immunotherapy^14^

2-[^18^F]-fluorodeoxy-d-glucose whole-body positron emission tomography/computed tomography (FDG-PET/CT) and 2-[^18^F]-fluorodeoxy-d-glucose whole-body positron emission tomography/magnetic resonance tomography (FDG-PET/MR) are well-established as part of the staging, therapy response assessment, clinical decision-making, and prognostication in patients with advanced CMM, head and neck cancer, sinonasal malignancies and particularly in SMM^2,12,15–17^. Limited data derived from heterogeneous sinonasal malignancy cohorts indicates a prognostic role of quantitative PET parameters for treatment response and survival outcome^15,18^.

The purpose of our study was to investigate the value of hybrid PET imaging for the assessment of treatment response in SMM patients undergoing ICI therapy. We hypothesized that hybrid PET imaging was useful in assessing therapy response in SMM patients treated with ICI.

## Methods

### Study design

**Figure 1:**
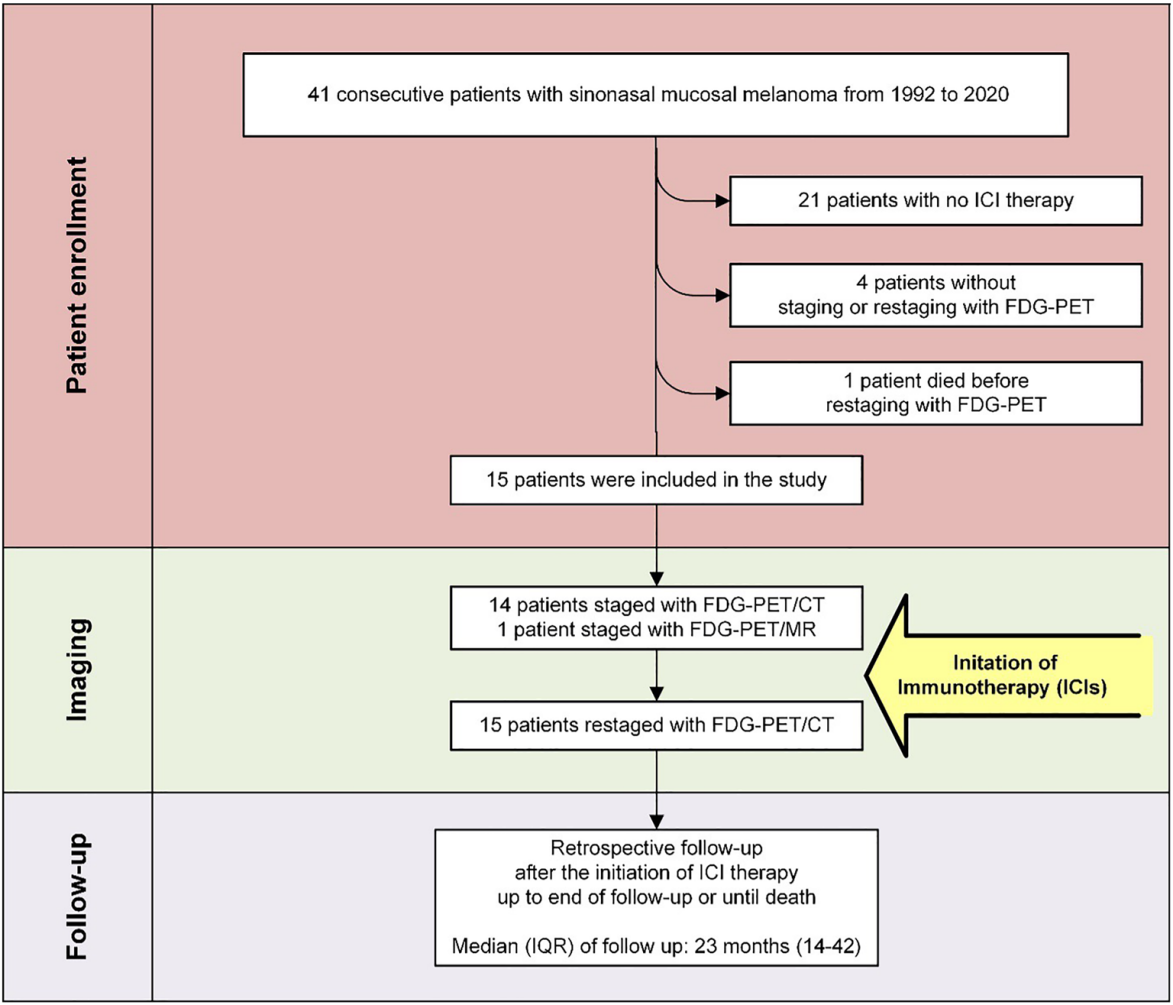
Study flow chart. Details of patient enrollment and study design. *FDG-PET/MR* 2-[^18^F]-fluorodeoxy-d-glucose positron emission tomography/magnetic resonance tomography, *FDG-PET/CT* 2-[^18^F]-fluorodeoxy-d-glucose positron emission tomography/computed tomography, *IQR* interquartile range.

This study received ethical approval from the Ethical Committee of the Canton of Zurich, Switzerland (KEK 2016-00162_Amendement), and was conducted in compliance with ICH-GCP rules and the Declaration of Helsinki. All individuals gave written informed consent to participate in the study. We retrospectively reviewed a consecutive cohort of SMM patients treated with ICI between March 2012 and October 2022 in the Department of Otorhinolaryngology, Head and Neck Surgery, in the Department of Radiation Oncology and the Department of Dermatology at the University Hospital Zurich, Switzerland. Every patient underwent staging with hybrid PET imaging before (ICI baseline) and at 3 months ± 4 weeks after the initiation of ICI therapy (ICI restaging) (Fig. 1). All patients and therapy plans were discussed at the multi-disciplinary dermato-oncological tumor board.

### Patient and tumor characteristics, surgical protocol and therapies

The following patient data and tumor characteristics were collected: age at diagnosis, gender, site of the primary tumor (nasal cavity vs. paranasal sinus), multilocular primary tumor (multiple sinonasal tumor foci), presence of lymph node metastasis or distant metastasis at primary diagnosis, initial staging (TNM category, AJCC 8th version)^19^, mutational status at initial diagnosis and lactate dehydrogenase (LDH) levels at ICI initiation. Local treatment details, including surgery [transnasal-endoscopic vs. open approaches (lateral rhinotomy)] and postoperative RT (median equivalent dose in 2-Gy fractions (EQD2)), as well as systemic treatment, were retrospectively assessed. Patients treated with anti-CTLA4, anti-PD1, or a combination thereof were included in the study. Immune checkpoint inhibitor therapy was applied (1) in the adjuvant setting, to reduce the risk of disease recurrence, (2) in locally advanced, unresectable tumors, or (3) in metastatic disease.

### Response assessment and disease-specific survival

Response assessment with FDG-PET/CT and FDG-PET/MRI after the initiation of ICI therapy was retrospectively reviewed and analyzed using the immunotherapy-modified Positron Emission Response Criteria in Solid Tumors (imPERCIST5)^20,21^. Response was rated as complete metabolic response (CMR), partial metabolic response (PMR), stable metabolic disease (SMD), or progressive metabolic disease (PMD). Patients with CMR, PMR or SMD were defined as responders to therapy; patients with PMD were defined as non-responders to therapy. Two independent physicians, who were dually board-certified in radiology and nuclear medicine and were blinded to the patient data, reviewed all FDG-PET/CT and FDG-PET/MR images. In case of discrepancy, a consensus decision was reached by case discussion.

### Impact of quantitative PET imaging parameters

For the sinonasal primary tumor, the following quantitative parameters of the staging PET were recorded: the maximum standardized uptake value (SUV_max_), metabolic tumor volume (MTV), and total lesion glycolysis (TLG). The selection of these parameters was based on previously published data and several decades of experience in a tertiary care hospital that serves as a national reference center for head and neck tumors and CMM^15,22,23^.

### Imaging technique

FDG-PET/CT was performed using a Discovery MI scanner (GE Healthcare, Waukesha, WI), Discovery 690 Standard scanner (GE Healthcare), Discovery VCT scanner (GE Healthcare), or Discovery ST scanner (GE Healthcare). FDG-PET/MR was performed using a 3 T PET/MR scanner (Signa PET/MR, GE Healthcare). According to our institution’s protocol, a standardized dose of 3.5 MBq of [^18^F]FDG per kg body weight (PET/CT) or 3.0 MBq per kg body weight (PET/MR) was injected until 2017, and from 2017 on, BMI-adapted body weight–dependent dosage protocols were used^24^. The CT included of a standardized protocol of high-resolution axial volume acquisition (0.6–1.0 mm) with reconstructions in the coronal and sagittal plane in the bone and soft tissue kernel with contrast enhancement of the sinonasal and neck region. For the sinonasal and neck MR, dedicated regionalized T2-weighted and T1-weighted pulse sequences with and without gadolinium-based contrast agent and with and without fat suppression were used^23^.

### Statistical analysis

Ordinal non-dichotomous variables are expressed as median and interquartile ranges 1–3 (IQR) and nominal non-dichotomous variables are expressed as modes and percentages. Comparisons of DSS for responders versus non-responders were analyzed using Kaplan–Meier survival analysis and stratified log-rank tests and are reported with hazard ratios (HR) and their 95% confidence intervals (CI). Univariate Cox proportional hazards regression analysis was performed to quantify the impact of ICI baseline quantitative PET parameters (SUV_max_, MTV, and TLG) of the primary tumor on DSS. Mann–Whitney U test was used to compare ICI baseline quantitative PET parameters of the primary tumor between responders and non-responders. A post-hoc power analysis was performed to evaluate the statistical power for calculations on SUV_max_, MTV and TLG. A *p*-value below 0.05 was considered statistically significant. Statistical analyses were performed using MedCalc Statistical Software version 19.6.4 (MedCalc Software bv, Ostend, Belgium).

## Results

### Patient and tumor characteristics

A total of 15 patients were included into the study. Patient and tumor characteristics are displayed in Tables 1 and 2**.** Primary SMM sites included the nasal cavity in 6/15 patients (40%) and both the paranasal sinus and nasal cavity in 9/15 patients (60%). The pathogenic mutational status of the primary SMM at initial diagnosis is presented in Table 3. Observed mutations included *NRASmut* (n = 2, 13%), NF1mut (n = 1, 7%) and *KRAS/KIT* (n = 1, 7%). Of note, two patients with initial *KRAS* and/or *KIT* mutation and acquired resistance to systemic treatment, switched their oncogenic driver to *NRAS* during systemic treatment. Likewise, one patient without evident driver mutation established an *NRAS* mutation after ICI therapy^25^. Systemic treatment was initiated because of gross tumor persistence due to unresectable disease (R2 resection) (n = 6, 40%), recurrence after initial resection and postoperative RT (n = 5, 33.3%) or synchronous distant metastatic disease (stage IV) (n = 4, 27%). In patients with synchronous distant metastatic disease at the time of ICI initiation, distant metastatic sites included liver (n = 1, 7%), lung (n = 1, 7%), bone (n = 1, 7%) and other visceral organs (n = 1, 7%). Five patients (33%) had ≥ 2 organs involved at the time of the ICI initiation.

**Table 1:** Patient and tumor characteristics.

Patient and tumor characteristics	n = 15
Age, years (median, range)	69 (39–84)
Sex, female (%)	9 (60)
Primary site (%)	
Nasal cavity	6 (40)
Paranasal sinus and nasal cavity	9 (60)
Unilocular vs. multilocular (%)	
Unilocular lesion	11 (73)
Multilocular lesions	4 (27)
Unresectable primary at ICI baseline (%)	
Yes	12 (80)
Stage at ICI treatment start (%, AJCC version 8)	
III	11 (73)
IVM1b	2 (13)
IVM1c	2 (13)
ECOG PS at ICI treatment start (%)	
≥ 1	1 (7)
Lactate dehydrogenase at ICI baseline (%)	
≥ ULN	2 (13)
Number of previous treatment lines (%)	
0	14 (93)
1	1 (7)
Presence of liver metastases at ICI baseline (%)	
Yes	1 (7)
Presence of bone metastases at ICI baseline (%)	
Yes	1 (7)
Presence of lung metastases at ICI baseline (%)	
Yes	2 (13)

*ECOG PS* Eastern Cooperative Oncology Group (ECOG) performance status (PS), *ICI* immune checkpoint inhibitor, *ULN* upper normal limits.

**Table 2:** Patients and tumor characteristics on an individual patient basis.

n	Sex	Age	Initial cT	Initial cN	Initial cM	Tumor epicenter	Surgical approach	Margin state	Postoperative RT	Indication ICI	Local disease control under ICI	Distant disease control under ICI
1	M	73	cT4b	cN0	cM0	Nasal cavity	Transnasal-transcribriform	R2, optic nerve, dura	Yes	Presence of distant metastases	Yes	Yes
2	F	72	cT4b	cN0	cM0	Nasal cavity	Sphenoethmoidectomy, tumor debulking	R2, orbit, dura	Yes	R2 resection	Yes	Yes
3	M	82	cT3	cN0	cM0	Ethmoidal sinus	Frontosphenoethmoidectomy	R0	Yes	Presence of distant metastases	Yes	Yes
4	M	85	cT4a	cN0	cM0	Ethmoidal sinus	Transnasal-transcribriform	R2, orbit	Yes	Recurrence, locally advanced and unresectable	Yes	Yes
5	F	49	cT4a	cN0	cM0	Maxillary sinus	Transfacial (maxillectomy)	Not applicable	Yes, brachytherapy	Recurrence, locally advanced and unresectable	Yes	Yes
6	F	71	cT3	cN0	cM0	Nasal cavity	Transfacial (rhinotomy)	R0	None	Presence of distant metastases	No	No
7	F	57	cT4b	cN0	cM0	Nasal cavity	Transfacial including extenteratio	R1	Yes	Recurrence, locally advanced and unresectable	Yes	Yes
8	F	64	cT4a	cN0	cM0	Nasal cavity	Transnasal-transcribriform	R1	Yes	Recurrence, locally advanced and unresectable	Yes	No
9	F	83	cT4a	cN0	cM0	Maxillary sinus	Sphenoethmoidectomy	R2, orbit	Yes	Presence of distant metastases	Yes	No
10	M	69	cT4b	cN0	cM0	Nasal cavity	Transnasal-transcribriform	R2, dura/ brain, multilocular	Yes	Recurrence, locally advanced and unresectable	Yes	No
11	F	43	cT4a	cN0	cM0	Nasal cavity	Sphenoethmoidectomy	R2, nasal floor multilocular	Yes	Adjuvant treatment, R2 resection	Yes	Yes
12	F	65	cT4a	cN0	cM0	Nasal cavity	Sphenoethmoidectomy	R1	Yes	R1 resection, locally advanced tumor	Yes	No
13	M	41	cT4a	cN0	cM0	Nasal cavity	Sphenoethmoidectomy	R1	Yes	R1 resection, locally advanced tumor	Yes	Yes
14	F	70	cT4a	cN0	cM0	Nasal cavity	Sphenoethmoidectomy	R2, maxillary bone, multilocular	Yes	R1 resection, locally advanced tumor	Yes	No
15	M	29	cT4a	cN0	cM0	Nasal cavity	Transfacial (lateral rhinotomy)	R2, maxillary bone, multiocular	Yes	Adjuvant treatment, R2 resection	Yes	Yes

The columns (1) local disease control and (2) distant disease control under ICI refer to the response at the first hybrid PET after initiation of ICI.

*ICI* immune checkpoint inhibitor, *RT* radiation therapy.

**Table 3:** Pathogenic mutations of the primary sinonasal melanoma tumor at initial diagnosis.

Mutation status	n = 15
NRASmut	2
NF1mut	1
KRAS	1
KRAS, KIT	1
BRCA2, PTERN	1
WT for the investigated genes	8
NA	1

### Treatment characteristics

All included patients underwent biopsy, tumor exploration and tumor resection under general anesthesia. The surgical approach comprised an endoscopic (fronto-)sphenoethmoidectomy in 7/15 patients (47%), an endoscopic transnasal-transcribriform resection with resection of the bony and/or dural anterior skull base in 4/15 patients (27%) and an open, transfacial approach with lateral rhinotomy in 4/15 patients (27%). Tumors were resected in piecemeal technique and the margin status was assessed with a circumferential mapping around the tumor. As depicted in Table 2**,** margin assessment revealed an R0 resection in 2/15 patients (13%), an R1 resection in 4/15 (27%) patients and an R2 resection in 8/15 patients (47%) (of note, in one patient the margin status was unavailable). R2 resection was due to (1) tumor infiltration of the orbital apex/optic nerve in one patient, (2) tumor infiltration of the orbital intraconal space in three patients, (3) tumor infiltration of the maxillary bone in two patients and (4) dural infiltration in two patients. Consecutively, postoperative RT was administered to 14 of the 15 (93%) patients. Thereof, one patient was treated with brachytherapy, one with protons and all other patients with photon-based intensity modulated radiotherapy (IMRT), implementing thermoplastic masks for immobilization and online image-guidance. The applied median cumulative dose was 66 Gy (IQR 62–66).

Thirteen patients (87%) were treated with systemic administration of ICI due to unresectable or metastatic disease, while two patients (13%) received ICI in the adjuvant setting a part of the primary treatment protocol. Systemic treatment included single-agent anti-PD1 (n = 5, 33%) or anti-CTLA4 (n = 3, 20%) and combined anti-PD1/anti-CTLA4 (n = 7, 47%). Fourteen patients (93%) were naïve to systemic treatment. At the time of the systemic treatment initiation, the majority of the patients were Eastern Cooperative Oncology Group (ECOG) performance status (PS) 0 (n = 4, 93%) and had LDH below the upper limit of normal (< ULN) (n = 13, 87%) (Table 1). In patients with unresectable/metastatic disease, overall response rate (ORR) was 69% (9/13 patients). Both patients treated in the adjuvant setting had later disease recurrence. At the time of analysis, six patients (40%) had completed their regular ICI treatment course and nine patients (60%) discontinued the ICI early. Overall, the median treatment duration was 9 months (IQR 4.5–17.5). The median follow-up duration was 48 months (IQR 17–79). Reasons for treatment discontinuation included progressive disease (n = 2, 13.3%), the patient`s will (n = 1, 6.7%) and toxicity (n = 6, 40%). The toxicity types were hepatitis grade 3 (n = 2, 13%, CTCTAEv5), arthritis grade 2 (n = 2, 13%, CTCTAEv5), neuropathy grade 3 (n = 1, 7%, CTCTAEv5) and colitis grade 3 (n = 1, 7%, CTCTAEv5). Detailed ICI treatment characteristics are provided in Table 4.

**Table 4:** Immune checkpoint inhibitor treatment characteristics.

Treatment characteristics	n = 15 (%)
Treatment setting (%)	
Adjuvant	2 (13)
Metastatic	13 (87)
Treatment type (%)	
Anti-PD-1/anti-CTLA-4	7 (47)
Anti-PD-1	5 (3)
Anti-CTLA4	3 (20)
Reason for treatment discontinuation (%)	
Progressive disease	2 (13)
Toxicity	6 (40)
Completed treatment	6 (40)
Patient’s will	1 (7)

### Response evaluation and disease-specific survival

**Figure 2:**
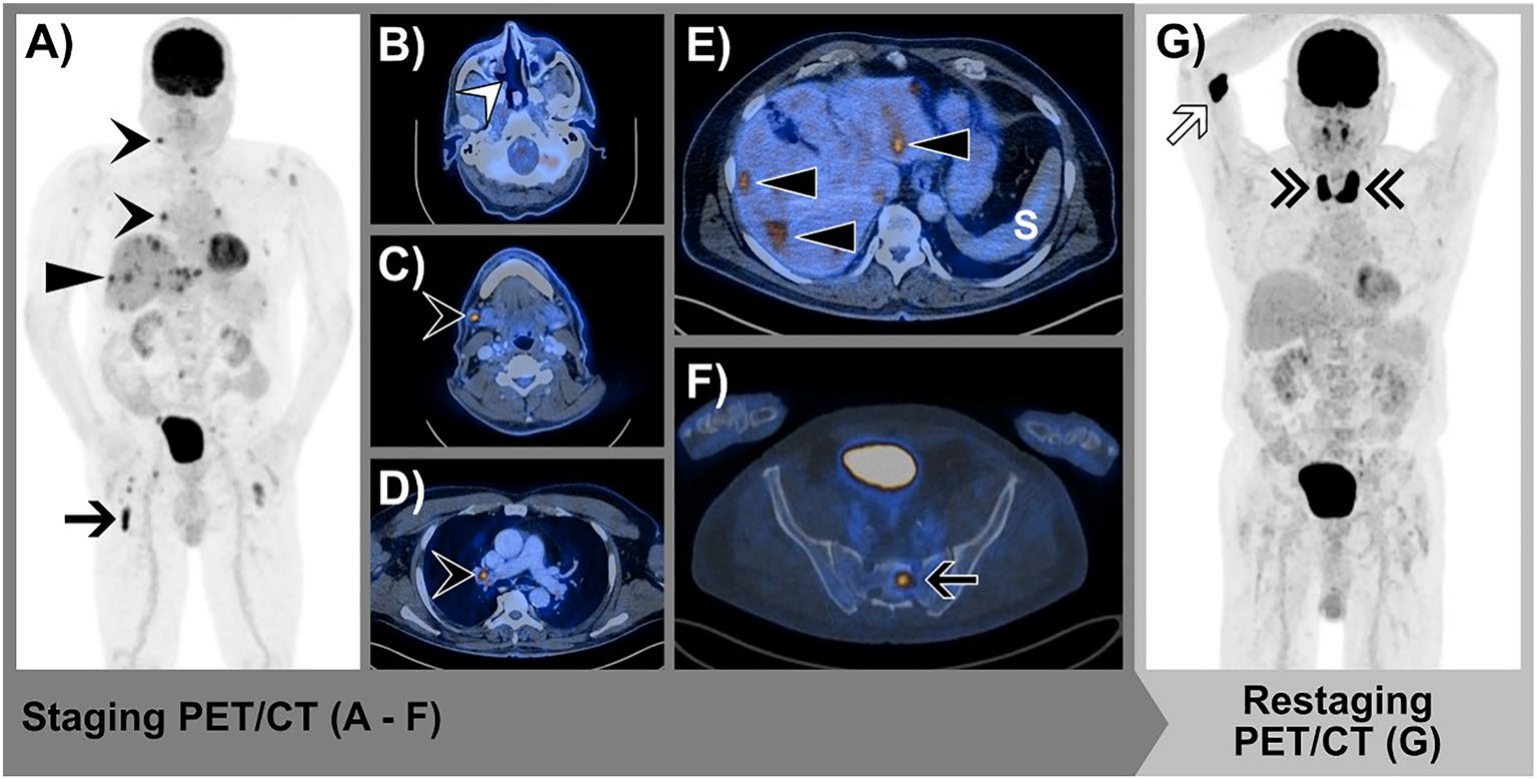
73-year-old male with SMM in the nasal cavity. FDG-PET/CT staging examination before the initiation of anti-PD-1-based therapy and after surgical resection and local RT: **(A)** Whole-body maximum intensity projection (MIP) image shows multiple cervical and mediastinal lymph node metastases (black arrowheads), multiple liver metastases (black triangle) and multiple bone metastases (black arrow). **(B)** Axial fused FDG-PET/CT depicted no local tumor persistence in the nasal cavity (white arrowhead). **(C)** Axial fused FDG-PET/CT with an example of a cervical lymph node metastasis (black arrowhead). **(D)** Axial fused FDG-PET/CT with an example of a mediastinal lymph node metastasis (black arrowhead). **(E)** Axial fused FDG-PET/CT with examples of liver metastases (black triangle). **(F)** Axial fused FDG-PET/CT with an example of a bone metastasis in the sacral bone. FDG-PET/CT restaging examination after the initiation of anti-PD-1-based therapy: **(F)** Whole-body maximum intensity projection (MIP) image shows CMR of all metastases. The newly appeared strongly increased uptake in the thyroid gland is consistent with immunotherapy-induced thyroiditis (double black arrowheads). FDG-extravasate at the cubital injection site (white arrow). *CMR* complete metabolic response, *FDG-PET/CT* 2-[^18^F]-fluorodeoxy-d-glucose positron emission tomography/computed tomography, *RT* radiation therapy, *S* spleen, *SMM* sinonasal mucosal melanoma.

**Figure 3:**
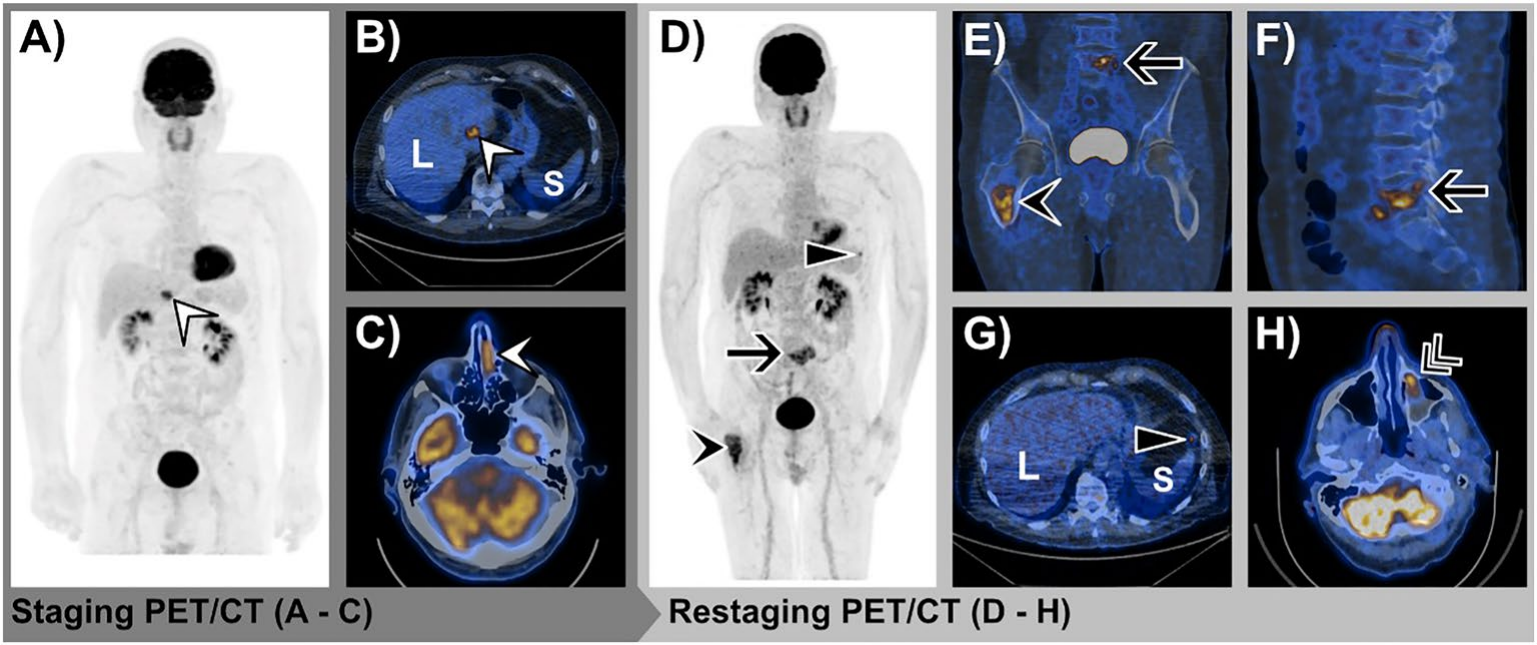
71-year-old female with SMM in the left nasal cavity. Initial FDG-PET/CT staging examination before the initiation of anti-PD-1-based therapy: **(A)** Whole-body maximum intensity projection (MIP) image shows an FDG-avid metastasis in the left lobe of the liver (white arrowhead). **(B)** Axial fused FDG-PET/CT confirms the FDG-avid liver metastasis (white arrowhead). **(C)** Axial fused FDG-PET/CT with the FDG-avid primary in the left nasal cavity (white arrowhead). FDG-PET/CT restaging examination 3 months after the initiation of anti-PD-1-based therapy shows PMD: **(D)** MIP image shows new FDG-avid soft tissue metastasis anterior to the spleen (black triangle) and a new bone metastasis in the fifth lumbar vertebral body (black arrow) and in the right proximal femur (black arrowhead). **(E,F)** Axial fused FDG-PET/CT with corresponding bone metastasis in the proximal femur (black arrowhead) and fifth lumbar vertebral body (black arrow). **(G)** Axial fused FDG-PET/CT with corresponding metastasis anterior to the spleen (black triangle). **(H)** New FDG-avid metastasis in the left maxillary sinus. FDG-PET/CT; 2-[^18^F]-fluorodeoxy-d-glucose positron emission tomography/computed tomography, *L* liver, *S* spleen, *PMD* progressive metabolic disease, *SMM* sinonasal mucosal melanoma.

**Figure 4:**
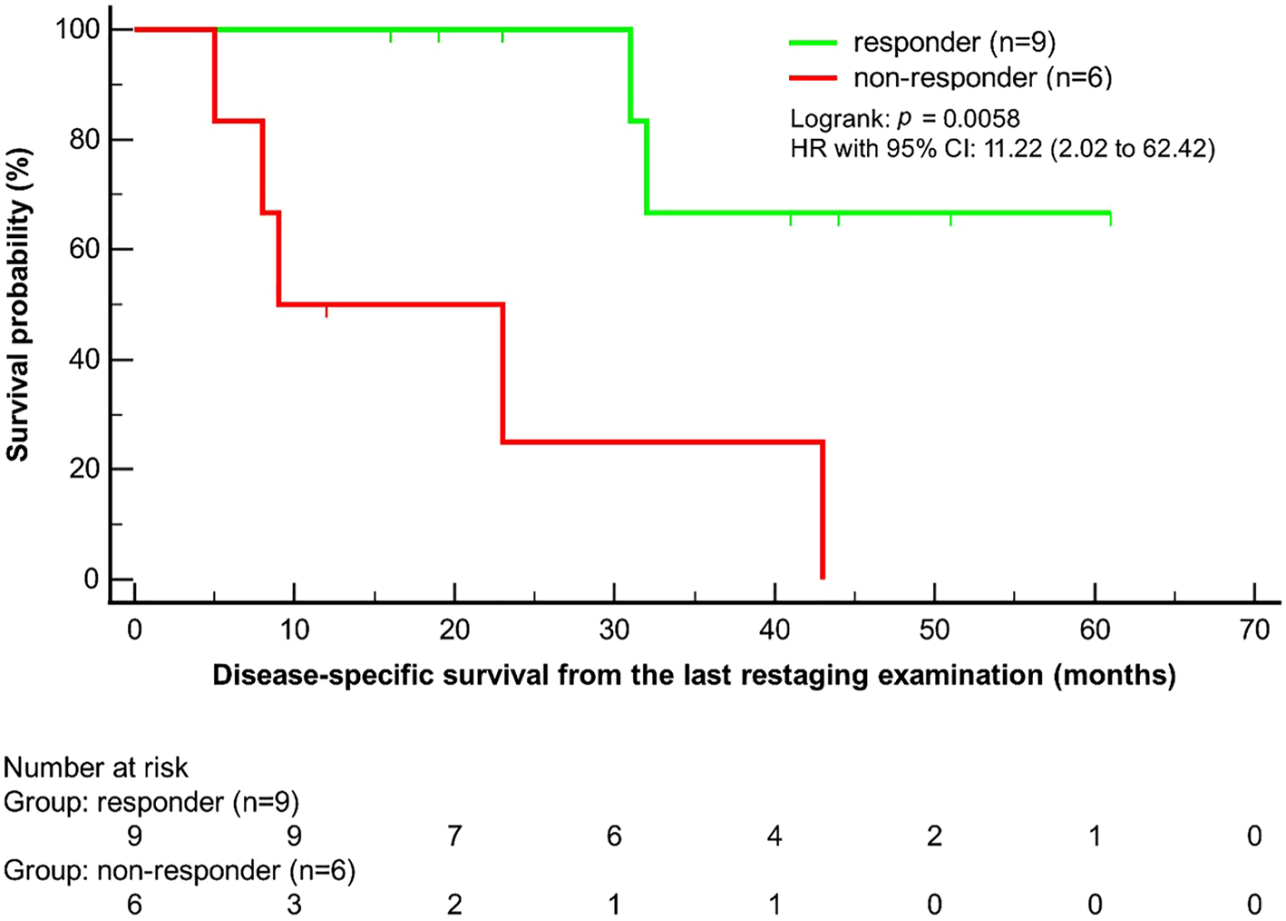
Disease-specific survival from the last restaging examination for responders compared with non-responders using imPERCIST5. The difference between the survival curves was significant (*p* = 0.0058). *CI* confidence interval, *HR* hazard ratio.

Fourteen (93%) patients underwent staging examination with FDG-PET/CT, whereas one (7%) patient underwent FDG-PET/MR. For restaging, FDG-PET/CT was conducted in all fifteen patients. The median time interval between staging and restaging examinations was 4 months (IQR 3–4). Upon restaging, seven (47%) patients had CMR, two (13%) had PMR, and six (40%) had PMD. No patient had SMD. Hence, response to therapy was demonstrated in 9/15 (60%) patients. (Figs. 2 and 3). In detail, as shown in Table 2, patients 1–5, 7, 11, 13 and 15 showed both, local and distant disease control under ICI treatment. In contrast, local but not distant disease control was seen in patients 8–10, 12 and 14 (Table 2). Disease-specific survival after the initiation of ICI therapy was significantly higher for responders compared to non-responders (χ^2^(1) = 7.62, *p* = 0.0058, HR = 11.22, 95% CI 2.02–62.42) (Fig. 4). Mean survival time was 51.2 months for responders and 20.2 months for non-responders. The median follow-up time from the restaging examination was 23 months (IQR 14–42).

### Impact of quantitative PET imaging parameters

**Figure 5:**
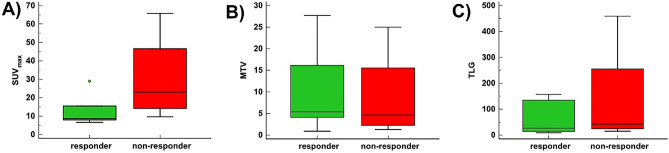
Boxplots of quantitative PET parameters measured in the primary tumor of patients for responders (n = 6) and non-responders (n = 4). No difference was found for **(A)** SUV_max_ (*p* = 0.088), **(B)** MTV (*p* = 0.669), or **(C)** TLG (*p* = 0.394). Of note, as explained in the *Results* section, only 10/15 patients (67%) had persistent/recurrent primary tumor on the PET scan before ICI start. *ICI* immune checkpoint therapy, *MTV* metabolic tumor volume, *SUV*_*max*_ standardized uptake value, *TLG* total lesion glycolysis.

10 of the 15 patients (67%) had local tumor persistence or recurrence at ICI baseline FDG-PET/CT or FDG-PET/MR scan, while there was no evidence of primary tumor persistence or recurrence in the other five patients. The median SUV_max_ of the primary tumor at ICI baseline was 12.6 (IQR 8.3–25.3), the median MTV was 5.2 (IQR 3.4–13.7), and the median TLG was 31.5 (IQR 17.1–113.9). No impact of these quantitative PET parameters on DSS was found (*p* = 0.639–0.964). Furthermore, no significant difference in quantitative PET parameters of the primary tumor were found between responders and non-responders [SUV_max_ (*p* = 0.088), MTV (*p* = 0.669), TLG (*p* = 0.394)] (Fig. 5). However, a post-hoc power analysis with a given “α” of 0.05 revealed that the sample size for SUV_max_, TLG and MTV was underpowered (28.0%, 10.6% and 3.5% respectively).

## Discussion

This retrospective single-institution study reflects the current multidisciplinary treatment approach for SMM patients. Surgical tumor resection forms the cornerstone of the primary treatment pathway. However, due to close proximity of vital anatomical structures and a frequent multilocular tumor growth, achieving clear surgical margins is challenging. Furthermore, the anatomic location is associated with considerable morbidity in case of extensive surgery. To improve local tumor control, and in accordance with current guidelines for the treatment of SMM, postoperative RT should be considered in patients with T3–T4 primary tumors with or without locoregional metastases^9,26,27^. Similarly, systemic treatment should be recommended in these patients after individual assessment of each case and upon multi-disciplinary discussion. Indications for ICI administration include resected SMM with high risk for local recurrence and presence of unresectable or metastatic disease. The combination of baseline and first restaging FDG-PET/CT after ICI initiation can help stratify patients with regard to DSS.

The advent of ICIs has led to a paradigm shift in the systemic treatment of advanced cutaneous melanoma. Nevertheless, there is currently limited data on the efficacy of ICIs in rare melanoma subtypes, such as mucosal melanoma. Hence, their role—particularly in the adjuvant setting—merits further investigation. As outlined by Flukes et al., ICI was initially mainly administered for unresectable disease and distant metastases, while later it was also incorporated in the adjuvant and neoadjuvant setting^28^. The indication for ICI therapy in our cohort reflects this transition of initiating a systemic treatment even in earlier melanoma stages, including locally advanced tumors with increased tumor thickness (T3–T4), with or without local and distant metastases. As previously shown, SMM patients with both, residual and recurrent local disease, revealed an inferior survival compared to those who achieved local control and were given ICI in the metastatic setting^28^. In a cohort study of patients included in the United States National Cancer Database no survival benefit in SMM patients undergoing ICI was seen, when compared to the current standard of care therapy, emphasizing that ICI might only be associated with improved survival in CMM^8^. One reason for that could be the difference in the genomic landscape of SMM compared to CMM^8^. Due to UV exposure, CMM have an increased tumor mutational burden, which is associated with response to ICI^29^. Moreover, CMM harbor more targetable mutations (especially BRAF V600 mutations) compared to SMM, leading to more treatment options besides ICI and therefore to an increase in OS. The SMM mutational landscape is rather marked by a low number of mutations, with most tumors being triple wildtype (no mutation in BRAF/NRAS/NF1) or *NRAS* mutated, therefore not being druggable^25^. In contrast, a recent large multicenter study on SMM by Lechner et. al. demonstrated the potential utility of further stratifying the T3 stage by sinus involvement and presented promising data on the benefit of immune checkpoint inhibitors for recurrent, persistent, or metastatic disease^9^.

In the last decade, hybrid PET imaging emerged as an alternative for the staging and restaging of sinonasal tumors, providing information on both metabolic activity and morphology, as well as on the presence or absence of metastases^15,22,30,31^. In patients with non-cutaneous melanoma, such as SMM, FDG-PET/CT is particularly valuable for staging, restaging and response assessment^16^. However, there have also been reports of FDG-negative, mainly small SMM tumors confined to the nasal cavity^15^. In a recent study investigating sinonasal malignancies, FDG-PET provided additional relevant clinical information beyond CT or MRI alone in 33% of examinations^22^. Similar findings were shown for CMM^32,33^. With regard to the role of FDG-PET/CT in predicting outcome in CMM patients treated with ICI, FDG-PET/CT imaging was shown to be superior to CT alone^12^. Additionally, Schank et al. reported that patients with CMR according to FDG-PET/CT may have a favorable outcome, even if ICI is discontinued^34^. In our SMM cohort, DSS distribution after the initiation of ICI therapy for patients with CMR or PMR was significantly better than for patients with PMD (Fig. 4). In a previous study, we could show that TLG in sinonasal primary tumors is an independent prognostic factor for achieving CMR after initial treatment^15^. However, in the current study, exclusively focusing on SMM, none of the quantitative PET parameters of the primary tumor had predictive value for the treatment outcome after ICI therapy (SUV_max,_ MTV and TLG). This might be attributed to several reasons, including the different treatment regimens, treatment lines, disease stage, metastatic sites, the addition of local RT at the primary site and also the comparably low overall number of patients.

In line with the current reference standard for first-line therapy in patients with SMM, the vast majority of our patients underwent surgical tumor resection followed by postoperative RT^7–9,11^. At initial presentation, patients frequently had advanced primary tumors, with infiltration of the orbit, the dura/brain or the maxillary bone. Multilocular tumor growth, a known risk factor for unfavorable outcome and non-R0-resection, was observed in 27% of patients (4/15 patients)^6^. This is worth mentioning, as the achievement of complete tumor resection with clear surgical margins is an important prognostic factor in SMM^35,36^. During the last decades, transnasal-endoscopic techniques have evolved and superseded open techniques in a substantial proportion of cases^37–39^. Traditionally, the ultimate goal of oncological surgery was to achieve an en-bloc resection with clear surgical margins and to avoid spillage of tumor cells^40^. However, owing to the complex anatomy surrounding the operation field, with close proximity to vital structures (e.g., optic nerve), this is often difficult to achieve by transnasal endoscopic techniques. Instead, tumors are resected in “piecemeal” technique, disassembling the lesions, with view of the borderline between the normal and infiltrated portions of the nasal mucosa^41^. This approach has been shown to be safe and effective, achieving equivalent results compared to open techniques, with less morbidity and decreased hospital stay duration^42^. In our cohort, an R0 resection was achieved in only 1/15 patient (7%), while an R1 (microscopic) and R2 (macroscopic) resection was achieved in 4/15 patients (27%) and 7/15 patients (47%), respectively (of note, in one patient the margin status was unavailable). Firstly, these findings reflect the locally aggressive and infiltrative character of SMM with frequent multilocular growth pattern. Secondly, modern multidisciplinary treatment algorithms opt for a maximization of preservation rates of vital structures, such as the orbit^43^. To improve local tumor control and following current treatment guidelines, postoperative RT should be considered in all patients with T3–T4 primary tumors with or without locoregional metastases^26,27^. However, data on the role of postoperative RT in SMM is scarce. In particular, the question whether an improved local control translates into a better OS is unclear and requires further investigation^44,45^.

Our patient cohort was overall small with a restricted statistical power. Especially the findings on quantitative PET parameters of the primary tumor (SUV_max,_ MTV and TLG) need to be interpreted with caution. However, it nevertheless represents one of the largest cohorts of SMM patients treated with ICI, undergoing staging and restaging hybrid-PET for therapy assessment. Other limitations include its retrospective nature, which incorporates a significant risk of bias, because no tumor board simulation was performed. Second, our cohort was heterogeneous, involving patients at different tumor stages, with various previous therapies and a heterogeneous indication for ICI. Third, a study duration of more than a decade harbors the risk of a natural evolution of knowledge over time and changing standards of therapy regimes. Fourth, pseudoprogression under ICI therapy is a known phenomenon and could have impacted the results of the response assessment.

In conclusion, FDG-PET/CT is a reliable instrument for the assessment of therapeutic response after the initiation of ICI in SMM. Treatment response shown on FDG-PET translates into a better DSS.

## Data Availability

Data can be made available upon reasonable request to the corresponding author.
